# Coping with Death Among Nurses in Ecuador: A Mixed-Methods Study

**DOI:** 10.3390/healthcare14050603

**Published:** 2026-02-27

**Authors:** Mónica Alexandra Valdiviezo-Maygua, Abigail Rivas-Lorefice, Alejandro Martínez-Granados, Daniel Puente-Fernández, Concepción Capilla-Díaz, Rafael Montoya-Juárez

**Affiliations:** 1 Programa de Doctorado de Medicina Clínica y Salud Pública, Universidad de Granada, 18071 Granada, Spain; mvaldiviezo@correo.ugr.es (M.A.V.-M.); abigailrivas@correo.ugr.es (A.R.-L.); 2 Departamento de Enfermería, Universidad Nacional de Chimborazo, Riobamba EC060150, Ecuador; 3 Servicio de Urgencias del Hospital Juan XXIII, 43006 Tarragona, Spain; alexmartinez0002@gmail.com; 4 Departamento de Enfermería, Universidad de Granada, 18071 Granada, Spain; danielpuentefdz@ugr.es (D.P.-F.); rmontoya@ugr.es (R.M.-J.); 5 Instituto de Investigación Biosanitaria (ibs.GRANADA), 18012 Granada, Spain; 6 Centro de Investigación Mente Cerebro y Comportamiento (CIMCYC), Universidad de Granada, 18011 Granada, Spain

**Keywords:** Bugen’s Coping with Death Scale, Ecuador, nurses, research methodology, palliative care, nursing, palliative medicine, terminal care, communication, comprehensive healthcare

## Abstract

**Highlights:**

**What are the main findings?**

- Ecuadorian nurses showed moderate coping levels with death, despite frequent exposure in clinical settings.
- Communication with dying patients and families was identified as a major professional challenge.

**What are the implications of the main findings?**

- Nurses in Ecuador experience significant emotional challenges when coping with death, implying a need for healthcare systems to recognise and address the psychological impact on staff.
- Providing ongoing education, emotional support, and training in spiritual and psychological care can strengthen nurses’ resilience, suggesting that investment in these areas may improve both nurse wellbeing and patient care quality.

**Abstract:**

**Background/Objectives:** Coping with death is an essential yet challenging aspect of nursing. In Ecuador, limited training and cultural factors may influence how nurses face the process of death and dying. This study aimed to explore nurses’ perspectives and highlight the degree of congruence between the numerical and discursive data provided by participants. **Methods:** A sequential explanatory mixed-methods design (QUAN → qual) using questionnaires and qualitative interviews was employed. The quantitative phase included 497 nurses who completed the Bugen Coping with Death Scale and the qualitative phase involved semi-structured interviews with 18 nurses. Quantitative data were analysed descriptively, while qualitative data underwent thematic analysis. Integration occurred at the methodological level—through the building of the qualitative data collection instrument—and at the levels of analysis and interpretation. **Results:** Nurses demonstrated moderate coping levels on the Bugen Coping with Death Scale. Although many reported being comfortable discussing death, qualitative data revealed substantial emotional distress and limited preparedness—particularly when facing their own mortality or the death of loved ones. Nurses expressed fear of suffering, sadness, and helplessness, especially when caring for dying children or young mothers. Communication with patients and families at the end of life emerged as a major challenge. Spirituality was identified as a key coping resource. **Conclusions:** Coping with death remains a complex and emotionally demanding process for nurses in Ecuador. Continuous education, emotional support, and training in spiritual and psychological dimensions of care are essential to strengthen nurses’ resilience and enhance the quality of care.

## 1. Introduction

Death is a natural and omnipresent phenomenon in human existence, representing the inevitable outcome of life. This event has a profound impact not only on the individual but also on their social environment, encompassing biological, social, cultural, and psychological dimensions [1]. In Western countries, advances in public health and medicine have led to a significant increase in life expectancy, although they have also contributed to distancing death from everyday life, rendering it something foreign or removed.

Confronting death requires strategies that are adapted to each individual’s experiences and sociocultural context. Poor management of this process can lead to fear, anxiety, reduced self-esteem, loss of religious beliefs, and dissatisfaction with life [2]. Healthcare professionals, particularly nurses, are routinely exposed to situations involving suffering and death, yet they too are affected by the social taboo surrounding death. Inadequate coping with these circumstances may result in fear, distress, emotional exhaustion, and compassion fatigue [3].

It is therefore essential that nurses—particularly those working in palliative care—develop coping strategies that enable them to provide comprehensive support in end-of-life situations, while also safeguarding their own mental wellbeing [1,4]. The perception of death is strongly shaped by the cultural and social context of each country. In Latin America, for instance, death occupies a prominent place within the culture, expressed through ceremonies and rituals.

Ecuador, with a population of approximately 17.5 million, reported a life expectancy at birth of 79.3 years for women and 73.9 years for men, ranking as the third highest in South America in 2019 [5]. This increase in life expectancy has led to a rise in age-related chronic conditions, highlighting the urgent need to develop palliative care services within the country. Between 2000 and 2020, the demand for palliative care rose by 44.1%, while the mortality rate increased from 62.4% in 2000 to 76.2% in 2019 [6].

However, the implementation of palliative care in Ecuador faces significant barriers, including a lack of training in advance care planning, limited infrastructure, restricted access to essential medicines, and an absence of specialised palliative care education for healthcare personnel [7,8]. The way in which healthcare professionals manage death plays a crucial role in promoting palliative care and supporting the wellbeing of healthcare staff in Ecuador.

Coping with death is usually understood within stress-coping theory as the changing cognitive and behavioural efforts used to manage the demands of patient death that feel overwhelming [9]. Strong coping-with-death skills are associated with less burnout and compassion fatigue and higher compassion satisfaction and wellbeing [10].

Instruments such as the Bugen Coping with Death Scale (BCDS) have been used to gain a better understanding of how nurses cope with death. The BCDS is a unidimensional tool developed by Bugen in 1980 to assess improvements in death competence [11]. It has been used to evaluate how nursing students cope with death [12,13] as well as nurses in a variety of clinical settings [2,14].

Exploring how healthcare professionals, specifically nurses in Ecuador, cope with death will enable strategies to be developed for better integration of palliative care in this country.

Qualitative methods make an in-depth exploration of the perceptions, experiences, and beliefs of professionals possible, allowing for a holistic and detailed understanding of coping with death within this specific context. This area has been examined in studies involving various healthcare professionals in other countries. Kurtgöz and Koç (2025) [15] investigated how nursing students cope with death. In Spain, Ruíz-Fernández et al. (2021) [16] conducted focus group sessions to explore the social factors that may influence coping with death among different healthcare professionals, including physicians, nurses, and clinical psychologists. Another study conducted in Mexico [17] analysed the coping strategies of oncology residents when facing death. To date, no qualitative studies have been conducted in Ecuador to explore this concept among practising nurses.

According to this, this study comprehensively examines how nurses in Ecuador cope with death, integrating quantitative and qualitative approaches to capture both coping competence and the experiential and contextual dimensions of this phenomenon. This will highlight the real situation of Ecuadorian nurses in relation to how they deal with the death of their patients, which will help guide clinical and educational policies.

This study adopts an innovative mixed-methods approach to examine how nurses in Ecuador cope with death, combining quantitative data (using the BCDS) with qualitative insights (through semi-structured interviews). The aim is to explore their perspectives and highlight the degree of congruence between the numerical and discursive data provided by participants. A mixed-methods approach enables the integration of quantitative and qualitative methodologies, thereby allowing for the identification of both similarities and differences in the findings obtained through each method [18].

In this sense, the aim of this study is to describe how nurses in Ecuador cope with death, explore their perspectives on the matter, and highlight the degree of congruence between the numerical and discursive data provided by participants.

## 2. Materials and Methods

A sequential explanatory mixed-methods design (QUAN → qual) was employed. The quantitative phase consisted of a descriptive study, while the qualitative phase followed a descriptive phenomenological approach. In qualitative research, the phenomenological approach focuses on understanding and describing the lived experiences of a particular phenomenon from an individual’s subjective perspective. The phenomenon under investigation was the experience of nursing professionals in Ecuador working in palliative care, including their perceived self-efficacy and level of perceived knowledge. Integration occurred at the methodological level—through the building of the qualitative data collection instrument—and at the levels of analysis and interpretation [19].

### 2.1. Sample

For the quantitative phase, a non-random, non-probability convenience sampling method was used. Participants were practising nursing professionals with at least six months of work experience.

Participants in the qualitative phase were randomly selected from among those who had indicated their willingness to be interviewed during the quantitative phase. In the qualitative study, the number of participants was determined by when thematic saturation was reached [20]. Saturation was determined as the point at which no new codes relevant to the study objectives emerged in consecutive interviews. After repeatedly analysing the transcripts, we observed thematic saturation in interview 16 and confirmed it with two additional interviews (*n* = 18). Sample heterogeneity was established based on the sociodemographic variables collected: sex, professional training, years of clinical experience, current healthcare service, previous work across different healthcare settings, and personal or professional experiences with individuals receiving palliative care.

### 2.2. Instruments

For the quantitative phase, an online questionnaire was distributed to participants via the LimeSurvey platform [21]. The questionnaire included a section on participant characteristics (sex, age, years of professional experience, and training in palliative care), as well as the Spanish-validated and Ecuadorian-adapted version of the BCDS. To avoid incomplete responses, the questionnaire did not allow participants to leave questions unanswered.

The BCDS comprises 26 items rated on a 7-point Likert scale, where 1 indicates ‘strongly disagree’ and 7 ‘strongly agree’. Total scores range from a minimum of 26 to a maximum of 182 [11]. The BCDS demonstrated a Cronbach’s alpha coefficient greater than 0.92 [11]. For this sample, Cronbach’s alpha was α = 0.943 (95%, CI = 0.937–0.947).

For the qualitative phase, a semi-structured interview was developed based on the items included in the BCDS. Additional questions were added to find out what changes had occurred in professionals regarding coping with death after the COVID-19 pandemic (Table 1).

The development process followed the recommendations set out in the Consolidated Criteria for Reporting Qualitative Studies (COREQ) checklist [22].

### 2.3. Data Collection Procedure

For the quantitative phase, professionals were contacted through the management boards of hospitals and primary care facilities, professional schools and associations, and care networks. Data collection was carried out between October and December 2022.

Participants in the qualitative phase were recruited from both hospital and community care settings. A date was arranged individually with each participant to conduct the interview.

At the beginning of each interview, the aims of the study were presented, the informed consent form was read aloud, and any questions or concerns were clarified. Participants were informed that the interview would be recorded, and oral consent was requested to confirm their agreement and participation. The reason for opting for verbal informed consent was for practical or logistical reasons, as the interviews were conducted via a videoconferencing platform.

The semi-structured interviews were conducted by MV, a researcher with specific training in qualitative research, between October and December 2023 via the Google Meet videoconferencing platform.

The average duration of the interviews was 53 min (range: 34–87 min). The interviews were transcribed in Spanish by researcher MV, and the technical review was carried out by researchers CC and RM. Fidelity verification was performed through systematic re-listening. The researcher who conducted the interviews and those who performed the technical review had no prior contact with the interviewees.

### 2.4. Data Analysis

For the quantitative analysis, percentages were reported for categorical variables (gender, university degree, contact with patients at the end of life, palliative care training, caring for someone at the end of life, and religious beliefs), and means and standard deviations were calculated for continuous variables (age, years of professional experience and BCDS scores). Previous studies employed three BCDS cut-off points for the total score of the scale, based on 33 and 66% percentiles [12,13], but not cut-off points for each item. After analysing the asymmetry of the BCDS items in the sample for this study, it was observed that the mean was −0.34, and so the following distribution of scores was decided upon: low (1–2), medium (3–5), and high (6–7) scores.

Data analysis was performed using SPSS version 28 [23] (IBM Corp., Armong, NY, USA, 2021).

For the analysis of open-ended responses, a thematic coding process was applied to participants’ answers, with the aim of generating a general understanding of the data based on the recurrent use of codes and patterns [24]. Interview results were organised into themes, subthemes, and code groups.

The themes were defined according to the structure of the interview guide: coping with one’s own death, coping with the death of others, and changes in coping with death. Subthemes corresponded to the interview questions. Codes were generated inductively. Each code was illustrated using verbatim quotes from participants, with the participant identifier shown in square brackets.

MV and CC performed coding in parallel, discussed discrepancies and agreed on a theme scheme among all authors with the first four interviews. They then applied this scheme to the rest of the interviews and organised periodic interviews to adjust the codes and define subthemes. Discrepancies were resolved by consensus among the different authors of the manuscript. Triangulation was performed between the different researchers. Qualitative data analysis was carried out using ATLAS.ti version 9.0.

### 2.5. Integration

Qualitative and quantitative data were integrated at two distinct points: during the sampling and data collection phase, and during the analysis and interpretation phase [19]. For the sampling and data collection stage, a ‘building’ integration strategy was followed, whereby the qualitative interview questions were developed based on the items of the BCDS.

The synthesis of the data was conducted by the researchers (DP and RM) and discussed and reviewed by the remaining authors (CC, AR, AM, and MV). The analytical approach used for the mixed-methods synthesis was triangulation, which involved classifying the merged results into categories of agreement, partial agreement, dissonance, and silence [25]. A side-by-side joint display was used to report the findings, linking the quantitative and qualitative results and generating meta-inferences [26].

Agreement was considered to be present when interview findings supported the quantitative survey results; partial agreement referred to situations in which results from different methods could not be regarded as fully equivalent without reservation; dissonance indicated contradictions between findings; and silence was identified when no correspondence existed between data obtained from the two methodologies.

### 2.6. Ethical Considerations

The present study complies with the fundamental ethical principles governing the responsible conduct of research involving human participants. Informed verbal consent was obtained from all participants. The study was approved by the Research Ethics Committee for the University of Granada (Ref. 2458/CEIH/2021).

## 3. Results

A total of 497 nurses participated in the quantitative phase of the study. Of these, 90.4% were women, and the mean age was 37.3 years (SD = 11.49). Participants reported an average of 11.47 years of professional experience (SD = 10.81), and the majority held a university degree (62.8%). A total of 82.7% stated that they had had occasional or frequent contact with patients at the end of life, while 12.1% reported having received training in palliative care during their professional career. Furthermore, 66.8% indicated that they had personally cared for someone at the end of life, and 67.4% identified themselves as practising Christians.

The total BCDS score was 98.71 (SD = 21.930). According to the scale’s own benchmarks, 1.8% of professionals (*n* = 9) demonstrated low coping, 62.4% (*n* = 310) moderate coping, and 35.8% (*n* = 178) high coping (Table 2).

### 3.1. Qualitative Study

The characteristics of the participants in the qualitative phase are presented in Table 3. The average number of years of clinical experience was 9.67 (±3.9) years. Of the sample, 88.8% were women, 61.1% held postgraduate qualifications (master’s degree), and the majority worked in hospital settings (66.6%).

### 3.2. Coping with One’s Own Death

Interviewees described death as an inevitable and natural human condition, irrespective of age, sex, social status, or religion. Some participants noted that everyone has a role to fulfil in life before dying.

*Death does not see age, sex, religion, or social status. It sees nothing—when it comes for us, it simply comes.*

*(I-17)*

*We all come into this world with a purpose, and no matter how harsh reality may be, once we have fulfilled that purpose, we must leave this world.*

*(I-16)*

Interviewees reported that death affects them emotionally, and that they must find a balance between remaining empathetic and not becoming overwhelmed by their feelings. Most stated that they do not feel prepared to die, particularly due to their young age, family responsibilities, or the need to care for their children.

*Psychologically or emotionally… yes, it hits me hard.*

*(I-5)*

*I’ve tried not to get too sentimental about it.*

*(I-2)*

*I don’t think I’m prepared for my own death, because when you’re a mother, you often stop thinking about yourself… and you think about your daughters.*

*(I-4)*

Some interviewees also expressed concern about unresolved matters and all the life experiences they would miss out on if they were to die. Above all, they feared the suffering their family would endure and the emotional void their absence would leave behind.

*The only thing I think is this: I just pray to God… not to have the kind of suffering we see in our patients. And of course, I’m very afraid.*

*(I-17)*

*As parents, we think about leaving our children on the right path. So yes, of course I’m afraid.*

*(I-12)*

*I don’t want to die because of the impact it would have on my family. And yes, I’m afraid.*

*(I-1)*

### 3.3. Coping with the Death of Significant Others

All interviewees agreed that losing a family member or close friend is a profoundly difficult experience, and that they do not feel adequately prepared to face it—especially when the person is very close or young. Most preferred not to think about the possibility of such an event occurring.

*Maybe it would be a little easier to cope if it were me who had to go, yes… but facing the death of my husband or children—I’d rather not think about it.*

*(I-1)*

*It depends on the family member. For example, if it were my parents, I don’t think I’d feel very prepared. But if it were my grandmother…*

*(I-3)*

### 3.4. Coping with the Death of Patients

The death of patients evokes feelings of sadness, pain, and helplessness among professionals. In particular, the frustration of being unable to provide the necessary support to the patient and their family leads to emotional exhaustion, which may manifest as depression and anxiety.

*It makes me feel very melancholic, very sad.*

*(I-11)*

*Sadness, helplessness… not being able to do more, you know?*

*(I-12)*

*You feel frustration, anger…*

*(I-18)*

Some interviewees inevitably saw themselves reflected in the dying situations they witnessed, which awakened feelings of empathy and gratitude for the wellbeing of their own families. They also reported experiencing a sense of fulfilment when death occurred under dignified and comforting circumstances.

*I always say that life is unique, and we have to make the most of every minute, every moment. We can’t say, ‘next year I’ll go for that walk’—no, I’ll take that walk today, because I don’t know if I’ll still be here next year.*

*(I-12)*

*I put myself in the shoes of the family member, the patient—because it could be someone in my own family in that situation one day.*

*(I-2)*

*It has been comforting to set aside the IV lines, the medication… and focus on the person who is dying, to tell them to go in peace, to go towards the light, that this is a journey—and to wish them well on their way.*

*(I-1)*

Participants highlighted the difficulties they face when communicating with patients in the final stage of life and with their families. One participant admitted to feeling an instinctive urge to prevent the patient’s death at all costs.

*I would say, ‘Dear God, give me the words to comfort them…’*

*(I-12)*

*We wanted to do everything in our power to stop them from dying—probably prolonging their suffering—but that’s the first thing that comes to mind: to stop it from happening.*

*(I-8)*

Turning to spirituality was also a commonly used strategy.

*I talk about the experiences I go through with my family.*

*(I-11)*

*When I’m in critical care, for example, I step away for a bit… I cry a little, wash my face, and… and go outside for a moment.*

*(I-7)*

*We all believe in a higher being—in my case, God—and sometimes it’s just about letting out the sorrow…*

*(I-16)*

Certain characteristics of dying patients influenced participants’ ability to cope with death—particularly in the case of paediatric patients, young mothers, or those with whom a special bond had been formed.

*With children… when it’s children, it’s something I just can’t get over.*

*(I-8)*

*Why do mothers have to die? Mothers shouldn’t die.*

*(I-12)*

*Some of my patients have been friends, and those deaths have really hurt.*

*(I-8)*

Interviewees also referred to other distressing situations that affected their ability to cope with death. These included family conflict during the patient’s final moments, witnessing the patient’s last breath, observing prolonged suffering without being able to alleviate it, the first patient death in their professional career, preventable deaths, and the suffering of good people due to illness.

*I remember we had a patient who just wouldn’t let go, and the days went by and we could see they were still holding on… And here in Ecuador, euthanasia isn’t legal, so we have to wait until the patient’s vital signs cease on their own.*

*(I-4)*

*It affects me more when it’s patients who come in with a sudden condition that could possibly have been treated in time.*

*(I-11)*

*It’s that feeling of helplessness. I had a chance to speak with the mother, and she said: ‘No, my daughter sinned—if she has to die, let her die.’ And they just let her die.*

*(I-7)*

### 3.5. Changes in Coping with Death

The vast majority of participants felt that being in contact with patients at the end of life had strengthened their own ability to cope with death. They emphasised the importance of making the most of life—enjoying it, spending more time with family, looking after their own health, seeking personal growth, and relying more on their religious faith.

*It’s still a difficult event, but it makes us a little stronger when it comes to facing death.*

*(I-4)*

*I believe in Jesus, and I believe—as the Word of God says—that ‘to live is Christ and to die is gain’, so every day I place my life in God’s hands.*

*(I-7)*

*Yes. I try to improve my lifestyle and take better care of myself emotionally.*

*(I-14)*

For participants, the death of patients often fostered a desire to improve the quality of care they provide in their daily practice. Many reported feeling stronger and more capable of facing patient deaths, while others acknowledged an increased sense of insecurity in this regard. The most significant change they experienced was a conscious effort to convey peace and reassurance to both patients and their families.

*We’ve become stronger, and we want to offer more support to the families—because death is hard, isn’t it? Sometimes it’s just not understood.*

*(I-9)*

*Sadly, our profession is so closely tied to death, isn’t it? So, from that day on I told myself, ‘no, we have to be strong, because they depend on us’.*

*(I-12)*

*I tried to remain as peaceful as possible—and of course, to do that I had to ask God to give me that peace, because I can’t fake it’.*

*(I-1)*

The experience of the COVID-19 pandemic was described by participants as particularly difficult and traumatic. Deaths during that period were marked by a sense of helplessness due to the lack of effective treatment, the absence of family members, difficult clinical decisions, and the moral conflict of caring for people who had not taken adequate precautions or complied with safety measures.

*There just wasn’t enough appropriate medication, because at that time we didn’t know what would actually help the patient.*

*(I-10)*

*The family found out about the death around eight hours later. And the worst part was that the patient had asked for them.*

*(I-3)*

*During COVID, we had teenagers—13, 14, 15 years old—coming in to be intubated, and then you had elderly patients aged 90, 92… And the doctors had to choose… It was really hard.*

*(I-7)*

*The people in these communities didn’t understand the social and public health implications of having a COVID-positive body outside of a coffin, you know? So they got infected… It was tough. We handled a lot of corpses.*

*(I-5)*

These experiences led participants to reflect on the fact that death affects people from all walks of life, and highlighted the importance of living with integrity, striving for personal growth, and seeking peaceful coexistence with others.

*What matters is the day-to-day—being a good person, helping when you can, and not being left with a guilty conscience at the end.*

*(I-16)*

*Before the pandemic, we just lived without thinking much about it. In fact, when my dad got ill, I started to realise that it could happen to any of us at any moment.*

*(I-1)*

Providing end-of-life care and developing empathy towards those in their care took on renewed significance. The pandemic, in particular, prompted professionals to reflect on the importance of funeral rites and spirituality.

*Knowing that they are still alive, that there’s still a beating heart… that they need to go with dignity. So yes, it has changed me—made me more considerate with those types of patients.*

*(I-11)*

*That’s also when you see people’s need to learn how to let go and to forgive—that’s the spiritual side that many lacked on their deathbed. I think we often fail to see beyond physical death; there’s also a spiritual death, because some patients couldn’t forgive, they cried… and then they died.*

*(I-10)*

### 3.6. Integration

Table 4 presents the results of the integration of quantitative and qualitative findings, indicating in the relevant sections whether there is agreement, partial agreement, dissonance, or silence between the two types of data.

In most cases, there was consistency between the quantitative and qualitative data; however, some partial dissonances and silences were also identified.

## 4. Discussion

This study aimed to describe the ways in which nurses in Ecuador cope with death using the BCDS, to explore their perspectives on the matter, and to highlight the degree of alignment between quantitative and qualitative data provided by participants.

For most of the issues explored, there was a strong degree of congruence between the BCDS scores and the discursive data obtained through qualitative interviews. However, the interviews revealed that death has a deeper emotional impact on Ecuadorian nurses than the scale might initially suggest. A lack of preparation to deal with death was evident—particularly regarding the death of loved ones. Notably, the nurses also demonstrated significant difficulties in communicating with patients who are nearing the end of life.

In terms of the quantitative data, Ecuadorian nurses showed moderate levels of coping skills in relation to death when compared to previous studies. The participants in the validation study by Galiana et al. (2017) [11], conducted among primary care professionals, reported higher scores on the BCDS. Nonetheless, the Ecuadorian nurses scored higher than those observed in studies involving nursing students [12,13].

Comparative studies among healthcare professionals between countries could help to understand variability in coping with death. In a comparative study among nursing students, Portuguese students reported higher scores in BCDS than Spanish students [27]. However, in a study that compared the perception of end of life among a sample of health science students in Spain and Bolivia [28], no statistically significant differences were observed between Spanish and Bolivian students using the BCDS. Further research should be conducted with healthcare professionals, specifically nurses, in Latin American countries.

The integration of quantitative and qualitative findings indicates that although a high percentage of nurses report feeling relatively comfortable discussing death, the interviews suggest that death has a considerable emotional impact on them. They must strive to strike a balance between demonstrating empathy towards patients and protecting their own emotional wellbeing. While empathy is essential to quality care, high levels of empathy without appropriate emotional management have been associated with compassion fatigue [29].

Furthermore, most interviewees reported not feeling ready to die themselves, particularly when this would involve experiencing a prolonged or painful illness. Another common concern among nurses was the suffering their families might endure when they are no longer present. Nurses often report feelings of fear and anxiety in relation to death, which have been associated with more negative attitudes towards end-of-life care provision [30]. Strengthening death coping skills through palliative care training may reduce fear of death, which could ultimately improve the quality of healthcare provision over the medium and long term [31].

The partial dissonance between quantitative and qualitative data in this matter should make us reflect on the accuracy of quantitative tools such as the BCDS for gathering nurses’ experiences with death.

Both the interviews and the questionnaire highlighted the value nurses place on living life to the fullest and making the most of the time available. This finding aligns with previous research suggesting that nurses tend to prioritise quality of life over its duration, even in end-of-life situations [32].

Quantitative and qualitative data were also consistent in relation to the significant emotional toll that the death of a loved one would have. Nurses expressed a clear reluctance to contemplate such an eventuality—an observation echoed in prior studies [33].

While many nurses reported feeling able to talk about their fears with family and friends, interviews also revealed that others prefer to manage these emotions individually. In this regard, communication and teamwork have been identified as protective factors when facing suffering and death [34]. Individual strategies such as meditation and mindfulness are also employed by nurses to manage fear [35]. Another study points to spirituality—as a search for meaning—as a fundamental strategy for coping with the death of patients [36]; however, further research is needed to clarify the role of spiritual beliefs in death-related coping.

Both the BCDS scores and the interview data reflect nurses’ efforts to convey peace and reassurance to patients and their families at the end of life. In this regard, the participants made frequent reference to both the patient’s spirituality and to the spiritual care provided by the nurses themselves. According to Evangelista et al. (2021) [37], Spanish nurses often struggle to deliver spiritual care due to personal beliefs or lack of training in this dimension. Similarly, Espinel et al. (2022) [38], in a study of spiritual care competencies in Latin America, found that many patients with advanced illness felt that their spiritual needs were unmet, as this dimension was often poorly integrated into holistic care. Some studies suggested that what nurses label “spirituality” may sometimes reflect religious ritual more than structured spiritual assessment and intervention [39,40]. It is worth considering what Ecuadorian nurses understand by spirituality, and whether there may be a conceptual overlap or confusion with religion, since most references in this study pertain to religious practices.

In relation to coping with the death of patients with certain characteristics, the vast majority of participants agreed that the most emotionally challenging deaths are those of paediatric or neonatal patients, closely followed by the deaths of young mothers. According to research by Shorey and Chua (2022) [41], this difficulty among paediatric nurses stems from the intense emotional impact and the lack of resources to cope with such complex experiences.

Lastly, although it was not a primary objective of this study, numerous references were made in the qualitative interviews to the severe impact that the COVID-19 pandemic had on how nurses in Ecuador cope with death. This period was experienced as extremely difficult and traumatic. Nurses of this study reported lack of effective treatment options, the absence of family members, ethically complex decisions, and the moral conflict of caring for people who had not followed health and safety guidelines. In this regard, the experiences reported by participants were similar to those described in other countries across Latin America and in Spain [42,43]. At the same time, the pandemic experience helped participants to realise that death affects people of all backgrounds, and underscored the importance of living with integrity, providing care until the end, and developing empathy for patients and their families. In a systematic review on attitudes towards death post-COVID-19, Gómez-Brufal et al. (2024) [44] highlighted the need for nurses to provide patients with a dignified death—something that was severely compromised during the pandemic.

### 4.1. Limitations

This study is subject to certain limitations. The use of convenience sampling raises concerns regarding selection bias and external validity. Random sampling was not feasible for the quantitative phase, as there was no centralised registry of nursing professionals in Ecuador from which to draw participants. Even so, the sample size achieved was sufficiently large to support the representativeness of the findings.

Regarding tools, the BCDS has been translated and adapted to the Ecuadorian context but, to date, the Ecuadorian version of the BCDS has not been published, so construct validity and cross-cultural equivalence of this version cannot be warranted.

Although previous studies employed three BCDS cut-off points for the total score of the scale [12,13], cut-off points were not established for each item. Readers have to consider that item cut-off points were decided based on BCDS items’ asymmetry.

As with other published studies on nurses and nursing students, most participants in the quantitative phase were women. This is representative of the nursing profession in Ecuador but could introduce a gender imbalance that should be considered when interpreting the results.

Secondly, qualitative participants were randomly selected from those who had indicated their willingness to be interviewed via the quantitative questionnaire. This self-selection may have introduced a degree of response bias.

### 4.2. Educational and Clinical Implications

The findings of this study underscore the need to strengthen nursing education on palliative care, coping with death and communication skills training across undergraduate and postgraduate curricula. Recent studies demonstrate how structured educational strategies—such as simulation-based learning or targeted training programmes—might improve coping with death in nursing students [45].

The results highlight the importance of organisational support mechanisms such as structured debriefings, teamwork, and psychological support, to address the emotional burden associated with patient death. Our results also point out that in Ecuador, there is a need for clearer conceptualisation and better integration of spiritual care into holistic nursing practice.

From an educational perspective, this would be possible by integrating a mandatory longitudinal module on palliative care and end-of-life care, both in undergraduate and postgraduate courses. It would be focused on training in, i.e., coping with death and grief, breaking bad news, family-centred communication and ethical decision-making at end of life. To ensure sustainability at the political level, it would be necessary to advocate for the inclusion of palliative care and emotional management skills in national nursing standards, as well as align curriculum reforms with accreditation requirements. Finally, it would also be necessary to encourage research funding for implementation studies that evaluate these strategies in the Ecuadorian setting.

## 5. Conclusions

This study explored how nurses in Ecuador cope with death, using the BCDS, and highlighted the degree of congruence between quantitative findings derived from the scale and qualitative insights obtained through semi-structured interviews.

Overall, the BCDS scores were broadly consistent with the qualitative accounts regarding nurses’ coping with death. The experience and perception of death appear to have a profound emotional impact on Ecuadorian nurses, who expressed a particular fear of suffering associated with dying, as well as concern for the impact their death might have on their loved ones.

From a clinical perspective, nurses reported in the interviews that difficulties emerged around communicating with patients about the end of life. From the perspective of our participants, spirituality seems to play a central role—not only as a personal coping strategy for nurses, but also as a crucial dimension of care for patients and their families. Additionally, many nurses reported particular emotional challenges when caring for paediatric or neonatal patients at the end of life, underlining the need for targeted emotional support and specific coping strategies in these cases.

The COVID-19 pandemic was described by our participants as a profoundly difficult and traumatic period. Nurses recalled feelings of helplessness due to the lack of effective treatment options, the absence of families at the bedside, ethically complex decision-making, and the moral tension of caring for patients who had not observed necessary safety measures. At the same time, the experience reinforced for many the universal nature of death, the value of living with integrity, the importance of providing dignified care until the very end, and the need for empathy towards patients and their families.

In summary, this study illustrates the complex nature of coping with death within the nursing profession in Ecuador. It underscores the need for ongoing training, updated resources, and a holistic approach to end-of-life care—one that integrates the spiritual and emotional dimensions of nursing practice.

## Figures and Tables

**Table 1:** Semi-structured interview guide.

**Coping with one’s own death and that of significant others**	What are your thoughts on death and the dying process?
	What has been your experience with coping with death?
	What are your expectations regarding your own death?
	How prepared do you feel to face your own death?
	What emotions and thoughts do you associate with your own death?
**Coping with the death of others**	How do you deal with the loss of significant people?
	What emotions or feelings are evoked by the death of patients?
	What attitudes have you adopted, and what resources have you used to cope with patients’ deaths?
	What situations affect your ability to cope with the death of your patients?
	Which type of patient do you find most difficult to care for when they are dying?
**Changes in coping with death**	What impact has being exposed to death had on you as a professional?
	What changes has this brought about in the care you provide to other patients?
	What lessons did you learn as a professional during the pandemic?

**Table 2:** BCDS scores.

		M	SD	% Low (1–2)	% Medium (3–5)	% High (6–7)
1	I have a good perspective on death and dying.	4.54	1.595	11.5	54.9	33.6
2	Death is a topic I can discuss and handle without difficulty.	4.23	1.586	16.3	61.8	21.9
3	I am aware of the full array of services from funeral homes.	4.34	1.655	16.3	57.9	25.8
4	I am aware of the variety of options for disposing of bodies.	4.15	1.723	19.7	55.9	24.3
5	I understand how to deal with the emotions that characterise the grieving process in each of its stages.	4.55	1.618	21.5	45.9	32.6
6	I feel prepared to face my death.	4.01	1.824	27.0	48.9	24.1
7	I feel prepared to face the illness process that could lead to my death.	3.81	1.753	27.6	52.9	19.5
8	I understand my death-related fears.	4.56	1.624	12.7	54.5	32.8
9	I am familiar with the arrangements and legal procedures that take place before a funeral.	3.84	1.823	29.0	49.9	21.1
10	I usually find it O.K. to think about death.	4.14	1.601	18.7	59.6	21.7
11	I can express my fears about death.	4.63	1.574	11.5	55.9	32.6
12	I can put my thoughts about death and dying into words.	4.42	1.454	10.7	64.2	25.2
13	I am making the best of my present life.	5.54	1.455	4.4	32.4	63.2
14	The quality of my life matters more than the length of it.	5.65	1.517	4.6	28.8	66.6
15	I can talk about my death with family and friends.	4.43	1.696	14.7	55.1	30.2
16	I know who to contact when death occurs.	4.66	1.669	12.3	50.5	37.2
17	I will be able to cope with future losses.	3.91	1.673	24.5	56.5	18.9
18	I feel able to handle the death of others close to me.	3.84	1.682	25.4	56.3	18.3
19	I know how to listen to others, including the terminally ill.	5.35	1.423	4.6	37.4	57.9
20	I know how to speak to children and adolescents about death.	4.22	1.58	17.1	59.2	23.7
21	I can spend time with someone at the end of life if needed.	5.28	1.429	4.8	42.1	53.1
22	I can help others with their thoughts and feelings about death and dying.	4.87	1.342	5.6	58.1	36.2
23	I would be able to talk to a friend or family member about their death.	4.59	1.454	10.5	60.4	29.2
24	I can lessen the anxiety of those around me when the topic is death and dying.	4.68	1.346	7.2	63.8	29.0
25	I can communicate with people who are at the end of their lives.	4.81	1.433	7.0	58.8	34.2
26	I am able to express my feelings and emotions to others before they or I die.	4.69	1.504	9.7	57.5	32.8

**Table 3:** Sociodemographic variables.

Code	Sex	Level of Education	Years of Clinical Experience	Current Work Setting	Previous Work Settings
*I* **-1**	F	Master’s in University Academic Management	15	Obstetric nurse and university lecturer	Obstetric facilities, gynaecological surgery, resuscitation, clinical care, outpatient care.
*I* **-2**	F	Bachelor’s in Nursing	6	ICU	Intensive care, emergency care.
*I* **-3**	F	Master’s in Nursing Unit Management	6	Haemodialysis	Haemodialysis, resuscitation, rural health.
*I* **-4**	F	Bachelor’s in Nursing	12	Intensive care	Community health, occupational nursing, haemodialysis, university lecturer, intensive care.
*I* **-5**	M	Bachelor’s in Nursing	2	Infectious diseases	Internal medicine, emergency care, clinical care.
*I* **-6**	F	Bachelor’s in Nursing	10	Internal medicine	ICU, emergency care, internal medicine.
*I* **-7**	M	Master’s in Gerontology in PC	10	Operating theatre or emergency care	ICU, emergency care, operating theatre.
*I* **-8**	F	Master’s in Clinical–Surgical Nursing	17	Obstetric facility	Surgery, trauma, internal medicine, obstetrics & gynaecology.
*I* **-9**	F	Master’s in Clinical–Surgical Nursing	23	Emergency care	Intensive care, inpatient care, emergency care.
*I* **-10**	F	Master’s in Surgical Nursing	14	Obstetric facility	Paediatrics, internal medicine, gynaecology, ICU.
*I* **-11**	F	Specialist in Primary Healthcare	11	Emergency care	Emergency care, intensive care.
*I* **-12**	F	Master’s in Health Management	20	Obstetric facility	Emergency care, burns unit, gynaecology.
*I* **-13**	F	Master’s in Clinical–Surgical Nursing	17	Obstetric facility	Emergency care, operating theatre, internal medicine, obstetrics & gynaecology.
*I* **-14**	F	Master’s in Public Health	19	PC	Internal medicine, paediatrics, PC.
*I* **-15**	F	Master’s in PC and Health	7	PC	Paediatrics, PC.
*I* **-16**	F	Bachelor’s in Nursing	5	Public health–Vaccination	PC, vaccination units.
*I* **-17**	F	Bachelor’s in Nursing	3	PC	PC, internal medicine, paediatrics, oncology, rural health.
*I* **-18**	F	Master’s in Public Health	10	PC	PC, vaccination units.

Abbreviations: I = interviewee; F = female; M = male; PC = primary care; ICU = intensive care unit.

**Table 4:** Main quantitative and qualitative results and their integration.

Quantitative	Qualitative	Integration
Over one-third of nurses reported having a positive perspective on death (Bugen1 = 33.6%, score 6–7), but fewer felt able to talk about it without difficulty (Bugen2 = 21.9%, score 6–7).	Interviewees stated that death is an inevitable and natural human condition, but that it affects them emotionally, requiring a balance between empathy and self-protection.	Partial dissonance: A high proportion of nurses feel relatively comfortable talking about death, but the interviews reveal significant emotional impact.
Nurses reported knowing how to deal with the emotions associated with grief (Bugen5 = 32.6%, score 6–7).		Silence: This topic was not addressed in the qualitative interviews.
A high percentage reported not feeling fully prepared to face their own death (Bugen6 = 27%, score 1–2) or the illness process that might lead to death (Bugen7 = 27.6% score 1–2).	Most interviewees said they did not feel ready to die. They feared the suffering associated with illness and death.	Agreement: Bugen scale scores align with qualitative findings.
Nurses felt they could understand (Bugen8 = 32.8%, score 6–7) and express (Bugen11 = 32.6%, score 6–7) their fears about death, and articulate their thoughts on dying (Bugen12 = 25.2%, score 6–7).	Most participants expressed concern about family suffering and the void they would leave. They also feared dying in pain.	Agreement: Both methods show clear concerns related to death.
Most nurses reported making the most of their lives (Bugen13 = 63.2%, score 6–7) and valuing quality of life over length (Bugen14 = 66.6%, score 6–7).	The vast majority emphasised the importance of enjoying life, spending more time with family, caring for their health, seeking personal growth, and turning to religion.	Agreement: Both data sources highlight the importance of living fully and meaningfully.
One-third of nurses reported being able to talk about their own death with friends and family (Bugen15 = 30.2%, score 6–7), and express their emotions regarding it (Bugen26 = 32.8%, score 6–7).	In some cases, participants shared experiences with family or colleagues; in others, they coped privately.	Agreement: Speaking to loved ones is one coping option, though not universal.
Most were informed about who to contact when someone dies (Bugen16 = 37.2%, score 6–7), but fewer were familiar with funeral arrangements (Bugen9 = 21.1%, score 6–7) or body disposition options (Bugen4 = 24.3%, score 6–7).		Silence: These topics were not discussed in the interviews.
A significant proportion did not feel prepared to face future losses (Bugen17 = 24.5%, score 1–2) or to manage the death of a loved one (Bugen18 = 25.4% score 1–2).	All interviewees agreed that loss is a painful experience and that they do not feel adequately prepared to cope with it. They prefer not to think about it.	Agreement: There is alignment in recognising lack of preparedness to face the death of others.
Most reported strong listening skills with terminal patients (Bugen19 = 57.9%, score 6–7) and being willing to spend time with them (Bugen21 = 53.1%, score 6–7), but fewer felt confident communicating with them (Bugen25 = 34.2%, score 6–7).	Participants highlighted difficulties in communicating with patients at the end of life and with their families.	Agreement: Interviews confirm communication as a challenge despite emotional availability.
A minority felt confident speaking about death with children and adolescents (Bugen20 = 23.7%, score 6–7).	Among the most difficult cases for participants were paediatric patients.	Silence: Interviews do not clarify whether the difficulty is linked to communication specifically.
Around one-third felt able to talk to family members about their death (Bugen23 = 29.2%, score 6–7), support others emotionally (Bugen22 = 36.2%, score 6–7), and reduce others’ anxiety when discussing death (Bugen24 = 29.0%, score 6–7).	Many participants actively sought to convey peace and reassurance to patients and families.	Agreement: The intention to reduce anxiety and provide comfort is evident in both datasets.

## Data Availability

The data presented in this study are available on request from the corresponding author due to ethical restrictions.

## Abbreviations

The following abbreviations are used in this manuscript:

I	Interviewee
F	Female
M	Male
PC	Primary Care
ICU	Intensive Care Unit
